# Genetic Engineering of the Biosynthesis of Glycine Betaine Modulates Phosphate Homeostasis by Regulating Phosphate Acquisition in Tomato

**DOI:** 10.3389/fpls.2018.01995

**Published:** 2019-01-10

**Authors:** Daxing Li, Tianpeng Zhang, Mengwei Wang, Yang Liu, Marian Brestic, Tony H. H. Chen, Xinghong Yang

**Affiliations:** ^1^College of Life Science, State Key Laboratory of Crop Biology, Shandong Key Laboratory of Crop Biology, Shandong Agricultural University, Tai’an, China; ^2^Department of Plant Physiology, Slovak University of Agriculture, Nitra, Slovakia; ^3^Department of Horticulture, Oregon State University, Corvallis, OR, United States

**Keywords:** glycinebetaine, *codA* gene, low phosphate stress, phosphate homeostasis, phosphate acquisition, tomato

## Abstract

Glycine betaine (GB), as a putative compatible substance, protects plants against the damaging effects of abiotic stresses. Phosphorus deficiency is one type of abiotic stress that is detrimental to plant growth. Maintenance of phosphate (Pi) homeostasis is crucial. This study demonstrates GB-regulated phosphate homeostasis in the tomato (*Solanum lycopersicum* cv. ‘Moneymaker’) transformed with the choline oxidase gene *codA* from *Arthrobacter globiformis*. The *codA*-transgenic lines displayed more resistance to low-phosphate stress. The data revealed that the wild-type plants were stunted and consistently retained less Pi than transgenic lines, especially when grown under low-phosphate conditions. This difference in Pi retention was attributable to the enhanced Pi uptake ability in the transgenic lines. The transgenic plants translocated more Pi into the plant cell due to the enhanced enzymatic activity of plasma membrane H^+^-ATPase and increased Pi/H^+^ co-transport, which improved Pi uptake. The differential expression of ‘PHO regulon’ genes further maintained intracellular Pi homeostasis. Furthermore, GB maintained a higher photosynthesis rate, thus increasing the production and translocation of sucrose via phloem loading to enhance plant response to low-phosphate stress. We conclude that GB mediates Pi uptake and translocation by regulating physiological and biochemical processes that promote adaptation to environmental changes in Pi availability. These processes eventually lead to better growth and development of the *codA*-transgenic lines. This finding will help to further elucidate the signaling mechanism of how GB perceives and transmits low-phosphate signals to alleviate Pi nutritional stress.

## Introduction

As an essential macronutrient, phosphorus is required for plant growth, development, and metabolism (Raghothama, 1999; Vance et al., 2003; Pandey et al., 2017). Phosphorus not only serves as the backbone for the biosynthesis of nucleic acids, membranes, phospholipids and ATP but also participates in many important biochemical pathways, including signal transduction, regulation of enzymatic activities, photosynthesis, and oxidative phosphorylation (Hamburger et al., 2002; Shin et al., 2004; Ai et al., 2009; Shen et al., 2011; Song et al., 2016). While the phosphorus content of the soil may be high, phosphorus deficiency can still arise due to precipitation and mineralization processes (Richardson, 1994; Yuan et al., 2017). Approximately 70% of global cultivated land is subjected to Pi deficiency (López-Arredondo et al., 2014). Thus, low phosphorus availability is a major constraint for plant growth and productivity (Mehra et al., 2016; Pandey et al., 2017). Inorganic phosphate is the only form of phosphorus that can be assimilated by plants (Chiou and Lin, 2011; Nussaume et al., 2011; López-Arredondo et al., 2014). Although Pi may fluctuate widely in soils, intracellular concentrations of Pi are strictly regulated to maintain homeostasis in plants (Chiou and Lin, 2011). To cope with phosphorus deficiency, plants have evolved a series of sophisticated strategies to maintain stable cellular Pi concentrations (Lin et al., 2009). These strategies involve physiological, biochemical and molecular responses, including the modification of root system architecture (i.e., reduction of primary root growth and the formation of more lateral roots and root hairs) (Watanabe et al., 2006; Lin et al., 2009; Lei et al., 2011; Mehra et al., 2016); the induction and secretion of acid phosphatases (APases) (Tian et al., 2003; Xiao et al., 2006; Wang X. et al., 2009; Mehra et al., 2017; Pandey et al., 2017), RNase (Löffler et al., 1992, 1993; Lin et al., 2009; Plaxton and Tran, 2011; López-Arredondo et al., 2014; Yuan et al., 2017), organic acid or protons (H^+^) (Otani et al., 1996; Yun and Kaeppler, 2001; López-Arredondo et al., 2014) into the rhizosphere contribute to the release of Pi from some organic sources; enhanced expression of high-affinity Pi transporter genes (Liu et al., 1998; Pandey et al., 2017) and establishment of differential photosynthate distribution between shoots and roots, resulting in increased root growth (Yun and Kaeppler, 2001; Gong et al., 2011; Yuan et al., 2017). In addition, Mehra et al. (2016) proposed that plant release Pi from membrane phospholipids through global membrane lipid remodeling under Pi deficiency. Mehra et al. (2017) also revealed the role of a novel rice purple acid phosphatases in improving plant utilization of organic-phosphorus. Recently, more adaptive strategies have been proved.

As is well-known, glycine betaine (GB) is one of the best-studied compatible solutes that enables plants to tolerate abiotic stress (Chen and Murata, 2002, 2008, 2011; Giri, 2011). Some studies have confirmed that GB has multiple functions in plant survival and growth, under both stressful and normal conditions (Yang et al., 2005, 2008; Park et al., 2007; Giri, 2011; Li et al., 2011; Masood et al., 2016; Kumar et al., 2017). However, few studies suggested that GB also interacts with mineral nutrition. Li et al. (2014) presented a new mechanism by which GB participates in salt stress tolerance. They indicated that GB acted as a cofactor of the Ca^2+^-CaM signal transduction pathway under salt stress. Wei et al. (2017) demonstrated that GB might regulate ion channel and transporters, resulting in high potassium and low sodium levels to enhance salt tolerance in transgenic plants under salt stress conditions. Nevertheless, the interaction between GB and phosphorus nutrition is still largely unknown. Several studies suggest that GB protects photosynthetic processes in stressful environments (Yang et al., 2007; Khan et al., 2009; Masood et al., 2016). In addition, phosphorus deficiency has immediate and direct consequences for photosynthesis (Plaxton and Carswell, 1999; Hammond and White, 2008). Therefore, we hypothesized that there may be a relationship between GB and phosphorus, and GB may play an important role in phosphate homeostasis.

In this study, we used *codA*-transgenic tomato plants, which were transformed with the choline oxidase gene *codA* from *Arthrobacter globiformis*. GB was accumulated *in vivo* as material to explore the mechanism used to enhance plant tolerance to phosphorus deficiency. Our results suggested that GB accumulation *in vivo* modulates phosphate homeostasis by regulating phosphate translocation and acquisition in tomato plants. Our findings shed light on the important role of GB in plant adaptation to low phosphate conditions and provide a new direction to explore the mechanisms by which GB modulates mineral nutrition.

## Materials and Methods

### Plants Materials, Growth Conditions, and Stress Treatment

The *codA*-transgenic tomato plants (L2, L3, and L4) and wild-type (WT) tomato plants (*Solanum lycopersicum* cv. ‘Moneymaker’) were used in this study. Our and others previous studies indicated the wide-type tomato plants are considered non-accumulators of GB (Park et al., 2004; Li et al., 2011; Kurepin et al., 2015; Kumar et al., 2017). The L2, L3, and L4 transgenic tomato plants were transformed with a gene (*codA*) for choline oxidase (Park et al., 2007). The seedlings (after germination) were grown in pots with sand containing the modified Hoagland’s solution. The seedlings were treated with 1.0 mM phosphorus (CK), 0.2 mM phosphorus, and 0.02 mM phosphorus (LP) for 15 days under sand-culture system. The nutrient solution was renewed every 3 days. The plants were grown in greenhouse at 25/20°C (day/night) with a photosynthetic photon flux density of 500 μmol m^-2^ s^-1^, a relative humidity of 65–70% and a photoperiod of 16/8 h light/dark.

### Extraction and Quantification of GB

The seedlings treated under normal or low-phosphate stress conditions for 15 days were used in this experiment. The GB content was measured following Rhodes et al. (1989) with some modifications. Leaf samples (four biological replicates were used in each genotype) were ground in methanol:chloroform:water (12:5:1) at 60°C for 30 min. The aqueous phase was fractionated by ion-exchange chromatography. After that, the GB fraction was eluted with 4 M NH_4_OH and dried on a rotary evaporator. Then, the preliminarily purified extract of betaine was analyzed by high-performance liquid chromatography (HPLC) and Millennium Chromatography Manager System Control software on a liquid chromatograph (SCL-10AVP; Japan) equipped with a Hypersil 10 SCX column.

### Determination of the Net Photosynthetic Rate (Pn)

Measurements of the Pn were performed at the same leaf position of the tomato plants in the morning between 9:00 and 11:00 at the seedling stage for different Pi concentrations. For each combination of genotype and Pi treatment, six biological repeats were performed. We used a portable photosynthetic system (CIRAS-3, PP Systems, Hitchin, United Kingdom) under the following conditions: 380 μl L^-1^ CO_2_, 800 μmol m^-2^ s^-1^ photosynthetic photon flux density, a leaf temperature of 25 ± 1°C and a relative air humidity of 60–70%.

### Determination of the Sucrose and Starch Content

The leaf content of sucrose and starch was determined using a kit from Nanjing Jiancheng Bioengineering Institute. For sucrose analysis, tomato plant samples (leaves) were extracted in 1 mL of 80% ethanol (v/v) for 10 min at 80°C in a water bath, and the samples were centrifuged at 4000 *g* for 10 min at 25°C. For destaining, 2 mg active carbon was added to the supernatant at 80°C for 30 min. Then, 1 mL 80% ethanol (v/v) was added and the samples were centrifuged at 4000 *g* for 10 min at 25°C and the supernatant was analyzed.

For starch analysis, tomato plant samples (leaves) were thoroughly ground in 1 mL 80% ethanol (v/v) and then treated for 30 min at 80°C in a water bath. Samples were centrifuged at 3000 *g* for 5 min at 25°C, and the residue was retained (the supernatant was discarded). Next, 0.5 mL of water was added, and the samples were incubated in a water bath for 15 min at 95°C. After cooling, the tissue residue was digested with 0.35 mL perchloric acid at 25°C for 30 min and oscillated 3–5 times. After that, 0.5 mL water was added, the samples were centrifuged at 3000 *g* for 10 min at 25°C, and the supernatant was analyzed.

The content of sucrose and starch were measured in three independent samples for each line. Statistical analysis was performed using Student’s *t*-test.

### Quantitative Measurements of Anthocyanin

The anthocyanin was extracted using a methanol-HCl method according to Rabino and Mancinelli (1986) with slight modifications. Tomato plant samples (0.2 g, three biological replicates for each condition) were incubated in 1 mL of acidic methanol (MeOH, HPLC quality) solution, consisting of 80% (v/v) MeOH, 0.16% (m/v) ascorbic acid, 0.16% (m/v) t-butyl hydroquinone, and 0.1% (m/m) HCl, with gentle shaking for 18 h at room temperature. After centrifugation at 12000 *g* for 2 min, 0.4 mL of supernatant was added to 0.6 mL acidic methanol and then the sample was filtered through a 0.22 μm filter before analysis. Extract absorbance was measured at 530 and 657 nm.

### Determination of the Activity of Sucrose Phosphate Synthase (SPS) and Sucrose Synthase (SS)

The enzymatic activities of SPS and SS for leaves were determined using a kit from Nanjing Jiancheng Bioengineering Institute. Three independent experiments were carried out per condition.

### Measurement of Fresh Weight, Total Phosphorus Content, and Pi Content

The tomato plants were treated under different Pi conditions for 15 days. After that plants were collected and then weighed.

The phosphorus concentrations of whole plants and the Pi content in the leaf, stem and root samples were determined colorimetrically by the molybdenum blue method, but the phosphorus concentration was measured after digestion in a mixture of H_2_SO_4_–H_2_O_2_ (Ames, 1966; Lei et al., 2011). For each combination of genotype and Pi treatment, three biological replicates were used.

### Plasma Membrane H^+^-ATPase Activity and Net H^+^ Flux in the Root Tip of Tomato Plants

The root plasma membrane isolation was performed following Yan et al. (2002) with some modifications. The root plasma membrane was stored at -80°C until analysis. The membrane protein concentration was quantified using the method Bradford (1976). Root plasma membrane H^+^-ATPase activity was measured according to the method of Yan et al. (2002). To assess the purification of H^+^-ATPase activity, H^+^-ATPase activity was expressed as the difference in activity between the presence and absence of 0.1 mm vanadate. A number of roots were collected for the extraction of plasma membrane, the total extractions were divided into three parts, and three replicates of total extracted plasma membranes of each treated plants of WT and transgenic plants were used for further determination of H^+^-ATPase activity. Finally, the plasma membrane extraction and the H^+^-ATPase activity determination experiments were repeated for three times.

The net fluxes of H^+^ were measured by Non-invasive Micro-test Technology (NMT) (NMT100 Series, Younger USA LLC, Amherst, MA, United States). For each line, six independent samples were measured. H^+^ flux measurements were recorded for 10 min, and H^+^ flux data were calculated with Mage Flux^1^.

### Quantitative Real-Time PCR Analysis

Total RNA was extracted from 100 mg of leaves and roots from WT and *codA*-transgenic tomato plants (L2, L3, and L4) using TRIzol reagent (TransGen Biotech; China). First-strand cDNA was synthesized from 1 μg of total RNAs using a reverse transcription system from TaKaRa. Q-PCR was performed using a SYBR^®^ PrimeScript^TM^ RT-PCR Kit (TaKaRa; China) in a 20 μL volume on the Bio-Rad CFX96 real-time PCR detection system. The quantitative real-time PCR experiment was repeated at least three times under identical conditions, using the housekeeping gene (actin) as an internal control. Primers used in the experiment are listed in Supplementary Table S1.

## Results

### GB Enhances Tolerance to Low-Phosphate in Transgenic Plants

**FIGURE 1:**
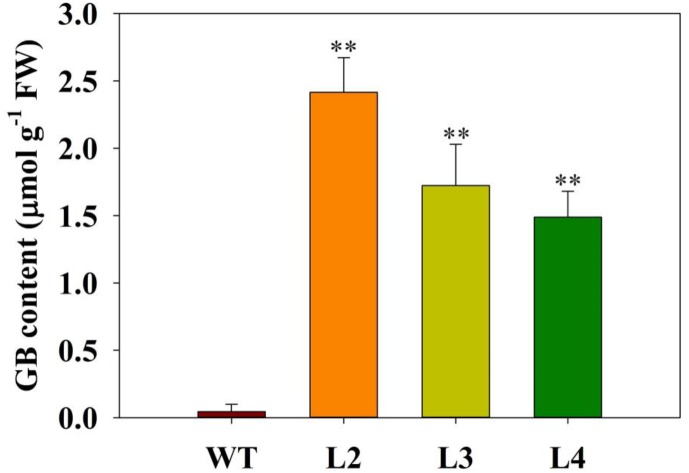
Levels of glycine betaine (GB) in the leaves of wild-type (WT) plants and three *codA*-transformed tomato lines (L2, L3, and L4). Plants were treated under normal or low-phosphate stress conditions for 15 days. Values represent the means ± SD of four replicates. Asterisks indicate significant differences compared with WT plants (Student’s *t*-test). FW, fresh weight; ^∗^*P* < 0.05; ^∗∗^*P* < 0.01.

**FIGURE 2:**
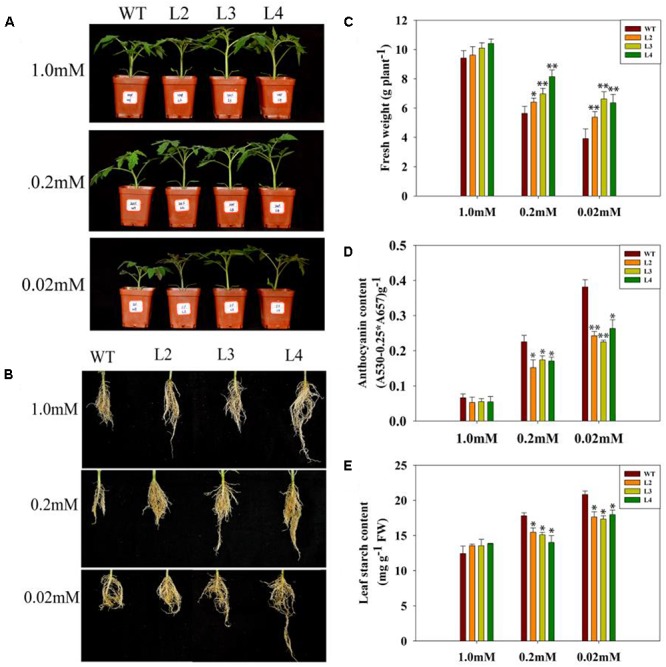
Growth phenotype of WT and transgenic lines under variable Pi conditions. **(A,B)** The shoot and root phenotypes of 15-day-old WT tomato plant and three *codA*-transgenic tomato lines under variable Pi conditions. Seedlings (after germination) were treated with 1.0 mM phosphorus (CK), 0.2 mM phosphorus and 0.02 mM phosphorus (LP) for 15 days under sand-culture system. Then, photos were taken. **(C)** The whole-plant biomass of WT and transgenic lines under the various Pi concentrations as described in **(A,B)**. 15-day-old tomato seedlings were collected for biomass analysis. Values are means ± SD. *n* = 12 for each genotype. **(D)** Anthocyanin content in the leaves of plants treated with 1.0, 0.2, 0.02 mM phosphorus for 15 days; seedlings were subsequently harvested for measuring anthocyanin content. **(E)** Starch accumulation of the 15-day-old WT and transgenic lines described in **(D)**. Values represent means ± SD of three replicates. Asterisks indicate significant differences compared with WT plants (Student’s *t*-test). FW, fresh weight; ^∗^*P* < 0.05; ^∗∗^*P* < 0.01.

Previous results showed that GB improved plant performance against environmental stresses (Ahmad et al., 2013). We wondered whether GB plays a role in tomato plants responses to low-phosphate stress. First, we measured the accumulation of GB in transgenic and WT tomato plants. Quantitative HPLC analysis demonstrated that the contents of GB in three independent transgenic lines ranged from 1.5 to 2.5 μmol g^-1^ FW, while GB was undetectable in WT plants (Figure 1). According to the result of Figures 2A–C, no significant differences in the phenotype or biomass were observed between the *codA*-transgenic tomato plants (L2, L3, and L4) and WT plants under normal conditions (1.0 mM). Under low-phosphate conditions (0.2 and 0.02 mM), the growth of transgenic plants and WT plants was inhibited (Figures 2A,B), but transgenic plants were significantly less affected than WT plants and showed the higher biomass of tomato plants (Figure 2C). These results indicate that the *codA*-transgenic tomato plants were more tolerant to low-phosphate stress than WT plants. To respond to low-phosphate stress, plants also induce anthocyanin and starch accumulation. Quantitative analysis showed that, under low-phosphate conditions, anthocyanin and starch content in transgenic tomato plants were less than in WT plants (Figures 2D,E). All the above indicate that GB affects multiple aspects of plant response to low-phosphate conditions.

### GB Participates in the Metabolism of Sucrose in Transgenic Plants

**FIGURE 3:**
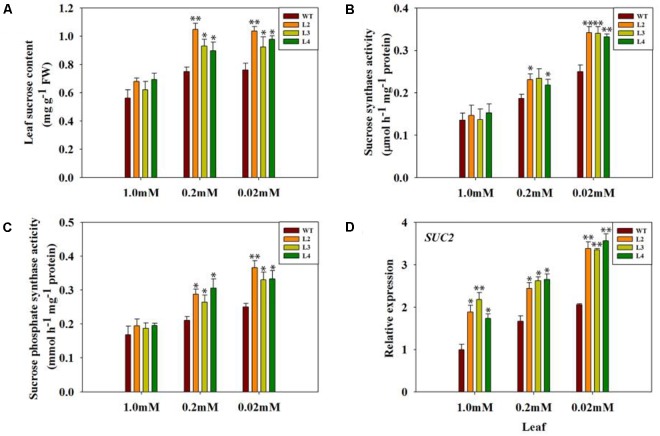
Sucrose concentration **(A)**, SS and SPS activity **(B,C)** and leaf *SUC2* gene expression **(D)** in WT tomato plants and three *codA*-transgenic tomato lines under variable Pi conditions. Seedlings (after germination) were treated with 1.0 mM phosphorus (CK), 0.2 mM phosphorus and 0.02 mM phosphorus (LP) for 15 days under sand-culture system. The seedlings were subsequently used for experimental analysis. Values represent means ± SD of three replicates. Asterisks indicate significant differences compared with WT plants (Student’s *t*-test). SS, sucrose synthesis; SPS, sucrose phosphate synthesis; FW, fresh weight; ^∗^*P* < 0.05; ^∗∗^*P* < 0.01.

Some studies have shown that sucrose may play an important role in the modulation of phosphorus metabolism under low phosphorus stress (Hammond and White, 2008; Lei et al., 2011). Therefore, we measured some physiological indexes of sucrose metabolism in WT and transgenic tomato plants to examine the role of GB in the adaptation response to low phosphate. The sucrose content in the leaves of *codA*-transgenic tomato plants was significantly higher than that of WT plants (Figure 3A). To further confirm whether the difference in sucrose content is caused by synthesis and/or transport, and the activity of sucrose synthase (SS) and sucrose phosphate synthase (SPS), which are enzymes that participate in synthesizing sucrose for sucrose loading into phloem, was determined in leaves of WT and *codA*-transgenic tomato plants (Figures 3B,C). Under Pi-deficient conditions, the activities of SS and SPS were significantly higher in *codA*-transgenic plants than in WT plants (Figures 3B,C). In addition, we also tested the expression of *SUC2*, which encodes a sucrose-proton symporter that is capable of transporting sucrose (Lloyd and Zakhleniuk, 2004), and the results indicated that *SUC2* was induced under low-phosphate conditions and that its expression in transgenic plants was far greater than that of WT plants (Figure 3D). These results suggested that GB may affect sucrose metabolism as a response to low-phosphate conditions.

### GB Maintains Higher Photosynthesis in Transgenic Plants

**FIGURE 4:**
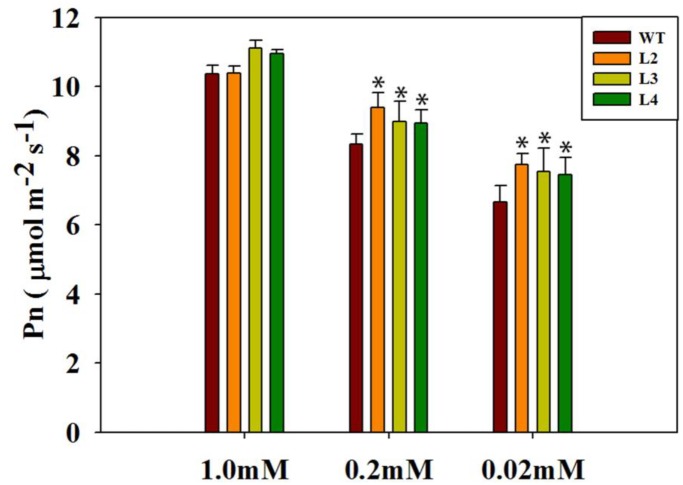
Photosynthetic rate (Pn) of leaves of plants grown with normal or low phosphorus. Seedlings (after germination) were treated with 1.0 mM phosphorus (CK), 0.2 mM phosphorus and 0.02 mM phosphorus (LP) for 15 days under sand-culture system. The photosynthetic rate was measured by the CIRAS-3. Values are means ± SD. *n* = 6 for each genotype. Asterisks indicate significant differences compared with WT plants (Student’s *t*-test). ^∗^*P* < 0.05; ^∗∗^*P* < 0.01.

To verify the elevated levels of sucrose in transgenic plants, we further analyzed photosynthesis under different Pi concentrations. Only under normal Pi levels (1.0 mM; Figure 4) did the transgenic plants appear similar to the WT plants. As the Pi concentration decreased, photosynthesis in the WT plants was severely inhibited. In contrast, photosynthesis has been maintained at an elevated level in the *codA*-transgenic plants even when they were grown under a very low Pi level (0.02 mM; Figure 4), indicating that GB may be involved in low-phosphate response, thus influencing photosynthesis.

### GB Affects Phosphorus Accumulation in Transgenic Plants

**FIGURE 5:**
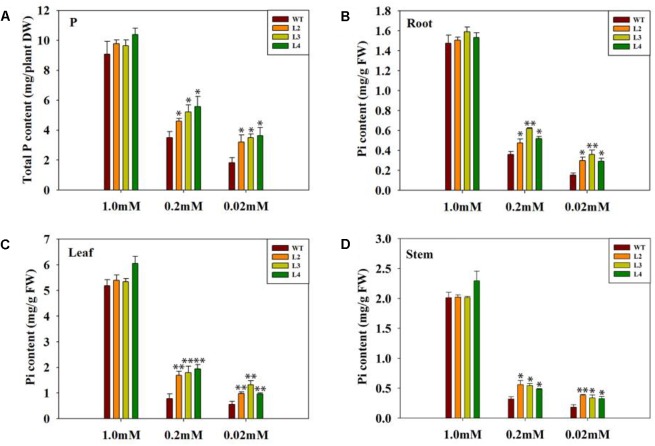
Comparison of total P and Pi content between WT and transgenic plants. Plants were grown under variable Pi conditions for 15 days. **(A)** Total phosphorus content of WT and transgenic lines under different Pi concentrations. Seedlings (after germination) were treated with 1.0 mM phosphorus (CK), 0.2 mM phosphorus, and 0.02 mM phosphorus (LP) for 15 days under sand-culture system. Seedlings were subsequently harvested for measuring phosphorus content. Values are means ± SD. *n* = 4 for each genotype. **(B–D)** The Pi content of the leaves, stem and root under normal Pi and low Pi conditions. Pi content was measured in harvested 15-day-old tomato plants. Values represent the means ± SD of three replicates. Asterisks indicate significant differences compared with WT plants (Student’s *t*-test). DW, dry weight; FW, fresh weight; ^∗^*P* < 0.05; ^∗∗^*P* < 0.01.

To sustain normal growth and development, it is important for plants to have enough phosphorus. We noted above that the transgenic plants adapted well to the low-phosphate condition. With the decreased Pi concentrations during growth, the total phosphorus in WT plants dramatically decreased, but the total phosphorus content in transgenic plants remained high (Figure 5A). To test whether the transgenic plants are better at absorbing and utilizing Pi, we measured the Pi content of various parts in both WT and transgenic plants. In all cases, the transgenic plants consistently retained more Pi compared with the WT plants (Figures 5B–D). These results suggest that GB may enhance the absorption of elemental phosphorus in transgenic plants under low-phosphate conditions.

### GB Activates Plasma Membrane H^+^-ATPase in Transgenic Plants

**FIGURE 6:**
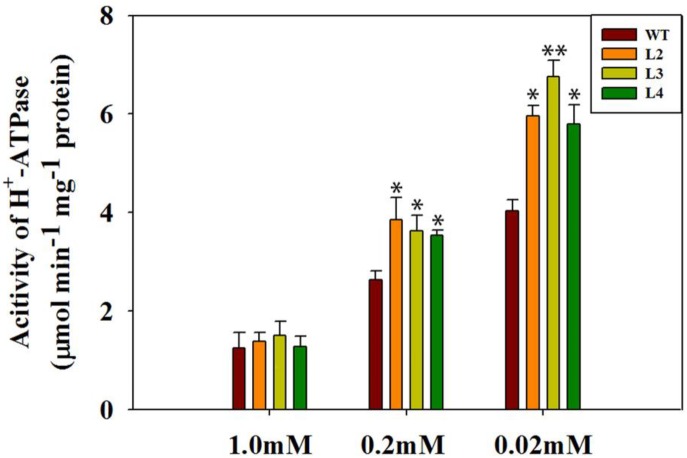
Comparison of plasma membrane H^+^-ATPase activity derived from tomato roots. Seedlings (after germination) were treated with 1.0 mM phosphorus (CK), 0.2 mM phosphorus, and 0.02 mM phosphorus (LP) for 15 days under a sand-culture system. Values are means ± SD of three replications per experiment, *n* = 6 for each genotype. Asterisks indicate significant differences compared with WT plants (Student’s *t*-test). ^∗^*P* < 0.05; ^∗∗^*P* < 0.01.

Transport of Pi across the plasma membrane is regulated by Pi/H^+^ co-transport stimulated by H^+^-ATPase activity (Raghothama, 2000). Phosphorus elemental analysis of tomato plants suggested that GB potentially functions in Pi absorption and utilization. To further support this hypothesis, we measured the activity of H^+^-ATPase in WT plants and transgenic plants. To evaluate the purity of root plasma membrane in tomato plants, the activity of various inhibitor-sensitive ATPases in the membrane fraction was analyzed (Table 1). As described in previous studies (Yan et al., 2002; Xu et al., 2012), our results showed that vanadate-sensitive ATPase occupied approximately 90% of the total activity in the plasma membrane, which indicated a highly purified plasma membrane. Afterward, plasma membrane H^+^-ATPase activity was analyzed in the whole root of WT plants and transgenic lines (Figure 6). Under normal conditions, no significant difference in H^+^-ATPase activity in the root plasma membrane was observed between the *codA*-transgenic plants and WT plants. However, the activity of root plasma membrane H^+^-ATPase in transgenic plants was clearly higher than that in WT plants under low-phosphate stress (Figure 6). Generally, protons (H^+^) in the plant cells are pumped out by the plasma membrane H^+^-ATPase (Zhang et al., 2011). These results partially suggested that the transgenic plants with enhanced H^+^-ATPase activity may have a stronger ability to secrete more H^+^ to regulate Pi absorption.

**Table 1:** The purity of plasma membrane isolated from tomato roots was analyzed by the activity of various inhibitor-sensitive ATPases in the membrane fraction.

	Na_3_VO_4_	KNO_3_	NaN_3_	Na_2_MoO_4_
1.0 mM	0.881 ± 0.02	0.091 ± 0.01	0.057 ± 0.05	0.022 ± 0.02
0.2 mM	0.874 ± 0.04	0.079 ± 0.02	0.038 ± 0.08	0.029 ± 0.05
0.02 mM	0.91 ± 0.02	0.066 ± 0.02	0.05 ± 0.03	0.032 ± 0.07


*The membrane was isolated from tomato roots subjected to normal and oxlow-phosphate stress conditions. The values are the means and SD of 10 replicates from two independent experiments.*

**FIGURE 7:**
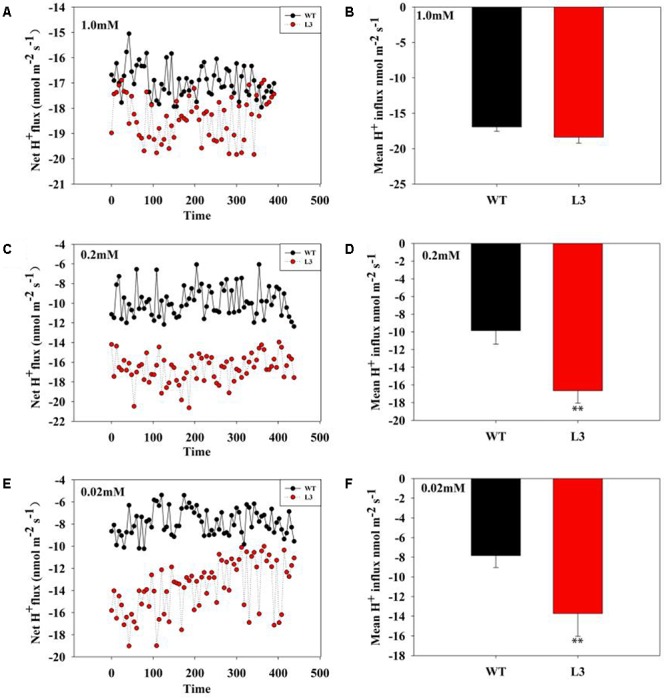
H^+^ flux in tomato roots under variable Pi conditions. **(A,C,E)** Transient net flux of H^+^ in the root elongation zone of WT plants and three *codA*-transgenic tomato lines under different Pi concentrations. A continuous flux recording over 10 min was conducted in a corresponding measuring solution (pH 6.0). **(B,D,F)** The mean rate of H^+^ flux within the measuring periods is shown. Values are means ± SD. *n* = 6 for each genotype. Asterisks indicate significant differences compared with WT plants (Student’s *t*-test). ^∗^*P* < 0.05; ^∗∗^*P* < 0.01.

The proton (H^+^) could couple with Pi to carry out Pi transport (Zhang et al., 2011). The relationship between Pi uptake and plasma membrane H^+^-ATPase activity was examined further by analyzing proton flux along the root tip of WT plants and *codA*-transgenic plants (Figure 7). We found that no significant difference in H^+^ influx was observed between transgenic plants and WT plants under normal conditions. Nevertheless, the H^+^ influx in *codA*-transgenic plants was significantly higher than WT plants at the root tip under low-phosphate stress (Figures 7C–F). These results indicate that GB accumulation *in vivo* increases the H^+^ influx, to promote Pi absorption in the root.

### GB Mediates the Expression of Pi Uptake and Translocation Related Genes in Transgenic Plants

**FIGURE 8:**
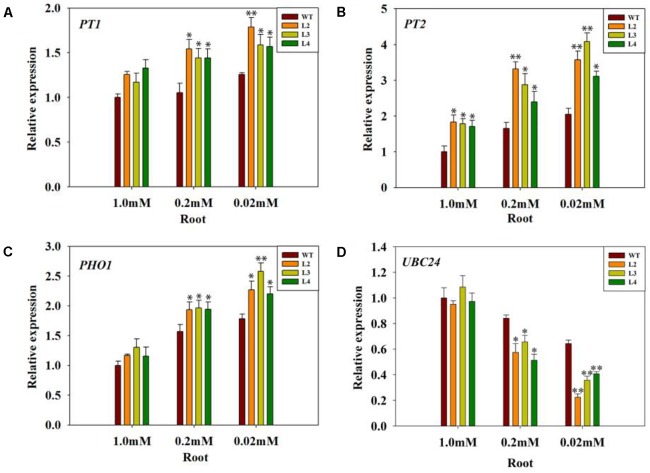
Quantitative analysis of gene expression of *PT1* **(A)**, *PT2* **(B)**, *PHO1* **(C)**, and *UBC24* **(D)** in the root of the WT tomato plant and three *codA*-transgenic tomato lines under variable Pi conditions. Seedlings (after germination) were treated with 1.0 mM phosphorus (CK), 0.2 mM phosphorus, and 0.02 mM phosphorus (LP) for 15 days under a sand-culture system. Seedlings were subsequently harvested for experimental analysis. Values represent the means ± SD of three biological replicates. Asterisks indicate significant differences compared with WT plants (Student’s *t*-test). ^∗^*P* < 0.05; ^∗∗^*P* < 0.01.

In addition, PHT1 transporters are responsible for Pi uptake from the soil (Hammond and White, 2008; Bucher and Fabiańska, 2016). *SlPT1* and *SlPT2*, members of the PHT1 family, are major high affinity Pi/H^+^ symporters in tomato whose expression is also highly induced by Pi starvation (Liu et al., 1998; Chen et al., 2014). Consistent with this observation, transcription of *SlPT1* and *SlPT2* was clearly induced in the *codA*-transgenic lines (Figures 8A,B), especially in the 0.02 mM treatment. In contrast, the same genes were expressed at a lower level in the WT plants. This result indicates that the induction of *SlPT1* and *SlPT2* in *codA*-transgenic lines was partially caused by GB-mediated.

The *PHO1* gene is involved in loading Pi into the xylem of roots (Hamburger et al., 2002). As shown in Figure 8C, the transcription level of the *PHO1* gene was evaluated in the roots of the *codA*-transgenic lines and WT plants. However, the transcription of *PHO1* was significantly enhanced in the *codA*-transgenic lines (Figure 8C), with the highest induction at 0.02 mM and the lowest under normal conditions. The *UBC24* gene was identified as *PHO2*, which negatively regulates Pi remobilization and uptake (Aung et al., 2006; Bari et al., 2006; Chiou et al., 2006; Zhou et al., 2017). Our results showed that the expression of *PHO2/UBC24* was clearly repressed under low-phosphate conditions (Figure 8D), and the repression level of *PHO2/UBC24* expression in the *codA*-transgenic lines was more marked than that in WT plants. These data indicate that GB *in vivo* may modulate the expression of some genes related to Pi uptake and translocation.

## Discussion

Plants require a large amount of Pi for their growth and development, but Pi levels are limited and constantly changing. To adapt to this nutrient stress, plants have evolved a strong Pi uptake and translocation capability (Liu et al., 2015). However, GB, as an important compatible solute, plays a vital role in various forms of abiotic stresses responses (Khan et al., 2009; Giri, 2011). Phosphorus stress is one type of abiotic stress. However, the relationships between GB and mineral nutrition remain unclear, especially for elemental phosphorus. Few studies indicated that the interaction between GB and mineral nutrients can be targeted to develop a tolerant phenotype (Masood et al., 2016). In this study, we confirmed the differential Pi uptake and translocation capacity between WT and transgenic plants and proposed a novel role that GB could alleviate low-phosphorus stress.

Phosphorus deficiency is detrimental to plant growth, development and metabolism. Several reports have shown that phosphorus deficiency leads to growth retardation and lowers the Pi level in plants (Ciereszko and Barbachowska, 2000; Lei et al., 2011; Xu et al., 2012; Su et al., 2015). In this study, although the *codA*-transgenic plants and WT plants both displayed a Pi-deficiency phenotype (Figure 2), including the level of internal phosphorus was reduced under low-phosphate stress (Figure 5), but the *codA*-transgenic plants were rendered more resistant to low-phosphate stress. We observed that the difference is not obvious in phosphorus content between WT and the *codA*-transgenic plants under normal condition and the *codA*-transgenic plants still maintained higher phosphorus content in the tissue compared with WT plants under low-phosphorus stress (Figure 5). This difference was probably due to that GB can affect the expression of some genes involved in Pi uptake and translocation and further promote Pi acquisition and translocation in low phosphorus stress condition (Figure 8). This further proved the notion that GB plays vital role in responding to low phosphate stress.

The *codA*-transgenic plants also display other characteristic responses to low phosphate levels, including reduced anthocyanin (Figure 2D) and starch (Figure 2E) accumulation. We speculated that these altered low-phosphate responses are caused by the GB accumulation in the transgenic plants (Figure 1). Numerous reports have demonstrated that GB plays a versatile and crucial role in imparting stress tolerance in plants (Takabe et al., 2006; Ahmad et al., 2013; Masood et al., 2016). Among these studies, none of them indicated that GB has any negative effects on plant growth and development under normal or stressful conditions (Ahmad et al., 2013). Therefore, we can infer that the transgenic plants suffered less under from smaller low-phosphate stress than WT plants.

Because the WT plants were smaller in stature, we suspected that this size difference could affect comparability of Pi measurements in the WT and transgenic plants. Thus, we next set out to determine the Pi uptake. Proton release into the rhizosphere is also a common adaptation to low phosphorus for enhancing phosphorus uptake (Raven and Smith, 1976; Lambers et al., 2006; Richardson, 2009). Generally, the increase in H^+^ secretion results from the activity of a plasma membrane H^+^-ATPase (Yan et al., 2002; Vance et al., 2003). The plasma membrane H^+^-ATPase plays an especially important role in the plant response to low-phosphate stress. Compared with WT plants, the transgenic plants exhibited higher activity of plasma membrane H^+^-ATPase low-phosphate conditions (Figure 6), suggesting that GB maybe involved in the low-phosphate response by activating root plasma membrane H^+^-ATPase to release protons. Several studies demonstrated that GB could positively affects complex proteins and antioxidative defense systems (Chen and Murata, 2011; Giri, 2011; Masood et al., 2016). In addition, our previous study (Wei et al., 2017) indicated that GB can regulate the H^+^-ATPase by enhance the expression of genes. We infer that GB accumulation due to the expression of choline oxidase in transgenic plants enhances the activity of H^+^-ATPase might associated with the protection to H^+^-ATPase and the enhancement of gene expression. However, the mechanism of how GB enhances enzyme activity remains to be further studied. The activation of the plasma membrane H^+^-ATPase may enhance the transport of phosphorus via establishing an electrochemical proton gradient that drives ion transport across the plant cell membrane (Haruta and Sussman, 2012; Yuan et al., 2017). Therefore, it is feasible to hypothesize that more H^+^ may be involved in Pi transport across the plasma membrane in the transgenic roots under low-phosphate stress, which facilitates Pi uptake. This inference is further supported by the enhanced H^+^ influx in the transgenic lines (Figure 7). Considering the stronger values of H^+^ influx in transgenic roots, we presume that more H^+^ do carry more Pi into the plant cell under low-phosphate conditions. This hypothesis is also confirmed by the phosphorus and Pi content measured in this paper (Figure 5). Wei et al. (2017) found that GB also regulated H^+^-ATPase activity in the *codA*-transgenic tomato lines under salt stress, to enhance Na^+^ exclusion and K^+^ uptake. In addition, growing evidence suggests that GB has a certain impact on ion absorption, including Na, K, Ca and others (Gobinathan et al., 2009; Alikhani et al., 2011; Wei et al., 2017). Therefore, we believe that GB can mediate Pi uptake by regulating proton circulation.

To further confirm that GB mediates Pi influx into plant cells, we measured the expression of *SlPT1* and *SlPT2* in the tomato roots, since the induction of *SlPT1* and *SlPT2* increased phosphate uptake (Liu et al., 1998). Interestingly, the results above showed that the transgenic tomato roots enhanced Pi uptake and root Pi content. In fact, the expression of *SlPT1* and *SlPT2* was also significantly induced in the transgenic lines in response to low-phosphate conditions (Figures 8A,B), showing that GB may modulate Pi uptake by directly up-regulating *SlPT1* and *SlPT2* expression. Pi homeostasis in plants depends not only on Pi influx into cells but also on Pi efflux. Proper distribution of Pi among the various plant tissues requires the loading and unloading of Pi in the xylem and phloem. The genes *PHO1* and *PHO2* have been identified as important to the control of Pi homeostasis (Hamburger et al., 2002; Aung et al., 2006; Bari et al., 2006). In addition, the *PHO1* gene has been demonstrated to transfer Pi into the xylem of roots (Poirier et al., 1991; Hamburger et al., 2002; Ribot et al., 2008). Then, we hypothesized that the improved Pi content in the stem and leaf of the *codA*-transgenic lines was partially caused by the differential expression of ‘PHO regulon’ genes. We noticed that the expression levels of *PHO1* in WT plants were clearly lower than that of transgenic lines even when they are grown under Pi-sufficient conditions (Figure 8C). In addition, reduced *PHO1* expression caused by *PHO1* mutations impedes Pi uptake (Liu et al., 2012), which also accounts for lower root Pi content of WT plants. The results provided evidence that GB may participate in the transport of Pi. In addition, down-regulated *PHO2/UBC24* alleviates the repression of Pi transporter genes and alters root growth and architecture to maximize Pi uptake (Aung et al., 2006; Bari et al., 2006; Sunkar et al., 2007; Wang Z. et al., 2009). The repression level of *PHO2/UBC24* was slightly stronger in the *codA*-transgenic lines (Figure 8D), which was consistent with their phenotype of higher expression of Pi transporter genes, stronger Pi uptake and translocation and better root development compared with WT seedlings. Previously, several studies have provided convincing evidence that the GB-accumulating transgenic plants have enhanced expression of stress-responsive genes (Kathuria et al., 2009; Chen and Murata, 2011; Giri, 2011), which might be a plausible explanation for GB-mediated genes related to Pi uptake and redistribution. These results further supported the speculation that GB plays an important role in Pi uptake and translocation under low Pi stress condition.

It is generally known that Pi deficiency has direct consequences for photosynthesis. Interestingly, GB has previously been reported to protect photosynthetic machinery in response to various type of environmental stresses (Bartels and Sunkar, 2005; Chaum and Kirdmanee, 2010; Chaum et al., 2013; Masood et al., 2016). In this work, we noticed that the transgenic plants exhibit better tolerance phenotypes than do WT plants, especially in the case of severe low-phosphate conditions (Figures 2A,B), and thus we naturally speculate that it may be closely related to the strong photosynthetic and metabolic processes in transgenic plants. As expected, physiological parameters showed that photosynthetic activity and fresh weight were significantly higher in transgenic plants compared with WT plants under low-phosphate conditions (Figures 2C, 4). Sucrose derived from photosynthesis serves not only as the major form of carbohydrate for long-distance translocation but also as a systemic signal of Pi signaling (Hammond and White, 2008; Zhang et al., 2014). Sucrose transport requires active loading, unloading and utilization of sucrose in the sink tissues. We found that low-phosphate stress enhanced the activities of sucrose synthesis enzymes, especially in the transgenic lines (Figures 3B,C). These data are consistent with sucrose content in both WT and transgenic plants (Figure 3A). In our work, a significantly higher *SUC2* expression in the leaves of phosphorus-starved transgenic plants was also observed (Figure 3D). *SUC2* encodes a sucrose-proton symporter that is responsible for sucrose loading into the phloem (Gottwald et al., 2000; Lloyd and Zakhleniuk, 2004). Wissuwa et al. (2005) speculated that the increased translocation of sucrose to the root may be driven by an increased root demand and that sucrose is likely to be utilized immediately by roots. Consequently, the transgenic lines with higher shoot sucrose concentration and better transport enable the plants to meet their need for root growth and to maximize Pi uptake, while lower sucrose biosynthesis and/or translocation attenuates plant response to low-phosphate stress in the WT plants. This is also consistent with the root phenotype results obtained under low-phosphate conditions (Figure 2B). Between root development and photosynthesis, a mutually beneficial relationship in the transgenic lines is established. Taken together, our results suggest that GB is involved in the response of plants to low-phosphate conditions via regulating leaf carbon allocation and sucrose transport to promote root growth.

## Conclusion

**FIGURE 9:**
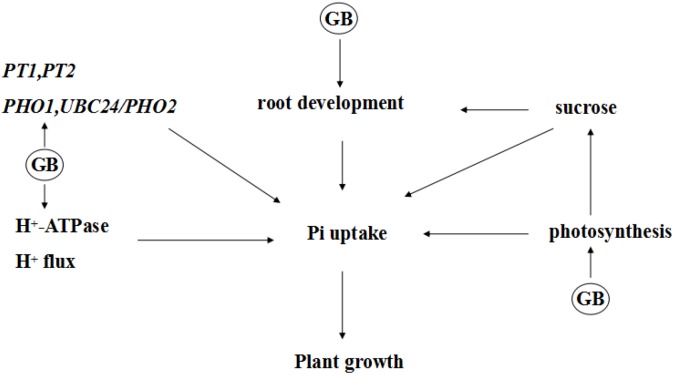
A possible model to show the probable mechanism of how GB regulates phosphate acquisition in transgenic tomato plants under low phosphate stress.

In summary, we investigated the potential mechanisms that GB mediates low Pi tolerance (Figure 9). We demonstrated that accumulated GB in transgenic tomato plants can alter the uptake of Pi; carbohydrate signaling; the expression of low-phosphate-response genes that are involved in Pi signaling, transport, mobilization; and the Pi balance between roots and shoots, which will ultimately maintain Pi homeostasis and help plant better adapt to low phosphate stress. A challenging task ahead is to identify the direct targets of GB and understand how GB perceives and transmits low Pi signaling to trigger plant Pi responses at the molecular level. Our result may benefit effort to enhance phosphate utilization efficiency of plant as well as to improve crop yield in low phosphate regions.

## Author Contributions

XY and TC designed the experiments. DL performed the experiments with the help of TZ and MW. DL and XY wrote the manuscript. MB and YL gave positive suggestion about this article. All authors read and approved the manuscript.

## Conflict of Interest Statement

The authors declare that the research was conducted in the absence of any commercial or financial relationships that could be construed as a potential conflict of interest.

## Footnotes

1 http://xuyue.net/mageflux
