# Supplementary material for: Genetic Engineering of the Biosynthesis of Glycine Betaine Modulates Phosphate Homeostasis by Regulating Phosphate Acquisition in Tomato
Source: Front Plant Sci. 2019 Jan 10;9:1995. doi: 10.3389/fpls.2018.01995 (PMC6335352; doi:10.3389/fpls.2018.01995)
Supplement: Supplementary file 1 [file Table_1.DOC]

**Supplementary Table S1. Sequences of primers (5’’) used for qRT-PCR analysis in tomato leaves or roots after 15-d treatment with normal and low-phosphate stress conditions.**

| Abbreviation | Primer sequence（5′-3′） |
| --- | --- |
| *ACTIN*-F | GGAACTTGAGAAGGAGCCTAAG |
| *ACTIN*-R | CAACACCAACAGCAACAGTCT |
| *PT1*-F | ATGCCTGAAACTGCCCGTTA |
| *PT1*-R | TTGGCTCCCTTTTGCTTCAA |
| *PT2*-F | AGAGGAAGCATCACAAGAAACT |
| *PT2*-R | ACCATTTTACAATAACACACTTGGC |
| *PHO1*-F | CATCAGCTCTGATAAGGTGGT |
| *PHO1*-R | ACAACCTGTGAAAAGCCCAA |
| *UBC24*-F | GCTTGACCATGGAACCCAAC |
| *UBC24*-R | TGGTTGATATGAGCATAAGAGGGA |
| *SUC2*-F | CCTACAGCGTCCCTTTCTCT |
| *SUC2*-R | GGATACAACCATCTGAGGTACAA |
